# From inhibition to recovery: metabolic rebound of *Microcystis aeruginosa* following pulse exposure to polystyrene nanoplastics

**DOI:** 10.1186/s12866-026-05260-9

**Published:** 2026-06-08

**Authors:** Ruiping Wang, Shizhong Yue, Jiafeng Yu, Li Jia, Cevin Tibihenda, Pingping Huang, Zhenghua Li

**Affiliations:** 1 College of Ecology, Resources and Enviroment, Dezhou University, Dezhou, 253023 PR China; 2 Shandong Key Laboratory of Biophysics, Institute of Biophysics, Dezhou University, Dezhou, 253023 PR China; 3 ISTO UMR7327, CNRS-Université d’Orleans-Brgm, Orléans, 45071 France; 4 College of Natural Resources and Environment, South China Agricultural University, Guangdong, 510642 PR China; 5 Tanzania Agricultural Research Institute, Dodoma, 1571 Tanzania

**Keywords:** Nanoplastics, Exposure, Post-exposure, Transcriptomic, Mechanism

## Abstract

Nanoplastics (NPs) are an emerging global environmental concern. While extensive research has focused on the toxicological effects of NPs on microalgae, the post-exposure consequences remain largely unexplored. In this study, *Microcystis aeruginosa* was exposed to 5 and 50 mg/L of polystyrene (PS) for 15 days, followed by a 15-day post-exposure period in PS-free medium. Exposure to 5 mg/L PS did not significantly affect growth, phycobiliprotein content, or oxidative stress responses. In contrast, 50 mg/L PS significantly inhibited algal growth and impaired photosynthetic capacity. Transcriptomic analysis revealed that this toxicity was underpinned by a broad downregulation of central energy metabolism, including photosynthesis, glycolysis, the tricarboxylic acid (TCA) cycle, and oxidative phosphorylation. Notably, following the removal of PS, *M. aeruginosa* exhibited a remarkable recovery capacity driven by metabolic rebound. This was manifested as a robust upregulation of previously suppressed pathways, particularly the pentose phosphate pathway and oxidative phosphorylation, facilitating a substantial, though potentially incomplete, restoration of physiological function. These findings highlight the metabolic resilience of *M. aeruginosa* and underscore the importance of considering post-exposure phases in ecological risk assessments of NPs.

**Supplementary Information:**

The online version contains supplementary material available at 10.1186/s12866-026-05260-9.

## Introduction

Global plastic production has significantly risen since the 1950s, with projections indicating that waste production will reach 12 billion metric tons by 2050 [12]. The primary concern is that only 14% of plastic packaging is collected for recycling, releasing vast quantities into the environment [20]. Plastics in the environment are fragmented into microplastics (MPs, 1 μm to 5 mm) and nanoplastics (NPs, less than 0.1–1 μm) via a combination of weathering processes [23]. Theoretical models, based on mass conservation, estimate NPs abundances to be ~ 10¹⁴ times higher than MPs [2]. NPs exhibit greater bioavailability due to their high specific surface area and hydrophobicity, enabling deep tissue invasion and causing more severe harm to aquatic organisms than MPs [43]. Consequently, NPs have emerged as a critical pollutant of global concern, demanding immediate research into their direct and indirect risks to aquatic ecosystems and human health.

Microalgae are crucial primary producers that help maintain ecological equilibrium within aquatic food chains [31]. The impact of NPs on microalgae has been investigated; however, conflicting conclusions existed. Some studies suggested that NPs inhibited microalgal growth by inducing oxidative stress and suppressing photosynthesis [32], while others indicated that NPs can promote microalgal cell growth and enhance the levels of chlorophyll, soluble proteins, and polysaccharides [19]. Therefore, the influence of NPs on microalgae involves multiple potential mechanisms that require further investigation. Moreover, while previous studies have predominantly focused on the toxicological effects of continuous NPs exposure, the post-exposure repercussions on microalgae remain poorly understood.

Real-word environmental studies revealed that NPs are non-uniformly distributed along coastlines. Furthermore, the transport and dispersion of plastic debris are influenced by a range of environmental drivers, such as wind dynamics and oceanic circulation [3], which lead to polluted areas with elevated plastic concentrations may alternate with less or no plastic waste [9]. This indicates that continuous NPs exposure on organisms cannot effectively predict the overall impact of NPs. The study by Copin et al. [8] revealed a persistent inhibitory effect of S-metolachlor on *Scenedesmus vacuolatus*, where growth suppression continued throughout the post-exposure period. In contrast, Nagai et al. [26] observed full post-exposure of *Pseudokirchneriella subcapitata* biomass during pretilachlor post-exposure period. Therefore, additional research is necessary to clarify the post-exposure impacts of NPs on microalgae and their associated mechanisms.

The microalga *Microcystis aeruginosa* (*M. aeruginosa*) is commonly found in freshwater environments and is a prevalent model organism in aquatic toxicity studies due to a number of advantageous characteristics including a rapid growth rate, sensitivity to contaminants and ease of handling [7]. Polystyrene (PS) plastics, representing one of the most prevalent polymer subclasses, serve as a predominant material in light industrial applications, particularly in the manufacturing of electronic and electrical components [40]. Therefore, this study employed *M. aeruginosa* to elucidate the influence of PS on algal during exposure and post- exposure. The evaluation encompassed key physiological and molecular responses, including growth dynamics and photosynthetic performance, oxidative stress responses and related enzyme activities, and transcriptional regulation. This study offers a more comprehensive insight into the potential risk of NPs to freshwater organisms, thereby establishing a foundation for understanding their broader ecological impacts on aquatic ecosystems and potential implications for human health.

## Materials and methods

### Experimental materials

*M. aeruginosa* (FACHB 905) was acquired from the Freshwater Algae Culture Collection at the Institute of Hydrobiology, Chinese Academy of Sciences (Wuhan, China). The cyanobacteria were cultured in BG11 medium under controlled environmental conditions: a light intensity of 2000 lx, a 12 h light:12 h dark photoperiod, and a constant temperature of 25°C. All experimental instruments were subjected to a steam sterilisation at 121°C for 20 min. Monodisperse PS microsphere with a nominal diameter of 80 nm and a solid content of 2.5% (w/v) were procured from Tianjin BaseLine Chromtech Research Centre (Tianjin, China). A 10 min sonication was performed on the PS solution prior to its use. The diameter and stability of PS in BG11 medium were measured using a Zetasizer Nano ZS instrument (Malvern, UK) and revealed an average diameter of 84.70 ± 0.75 nm, and a zeta potential of -33.96 mV ± 0.40 (Fig. S1).

### Experimental setup

After being centrifuged and washed, logarithmic-phase *M. aeruginosa* cells were resuspended in 600 mL of BG11 medium, achieving an initial cell density of 1.7 × 10⁶ cells/mL. The cultures were subjected to PS exposure at concentrations of 5 and 50 mg/L, with a control group without PS supplementation. Although MPs concentrations in some polluted aquatic environments, such as Lake Taihu, have been reported to reach levels of 1.86–14.18 mg/L [34, 41], these values typically refer to larger MPs particles with a diameter greater than 1 μm. For sub-micron NPs, the actual mass concentrations in natural waters are generally in the range of ng/L to µg/L [1]. Given that the primary objective of this study was to investigate the mechanistic responses and metabolic resilience of *M. aeruginosa* under NPs stress, we employed 5 mg/L as a lower exposure concentration and 50 mg/L as a worst-case, high-exposure scenario, rather than as direct representations of typical environmental background concentrations. Furthermore, this study was designed to assess the integrated toxicity of NPs (considered as a holistic physico-chemical entity) on *M. aeruginosa;* therefore, the experimental setup included only NPs exposure groups and blank controls, without a separate MPs control group, as the primary objective was to evaluate the overall toxicity of NPs.

Each treatment was repeated three times, and all flasks were incubated for 15 days. Upon completion of the PS exposure, the algal cells were subjected to centrifugation for harvesting and transferred to clean 600 mL PS-free BG11 medium for further culture for 15 days. This procedure does not guarantee the complete removal of PS that may be firmly adsorbed onto the cell surface or internalized. Additionally, we did not assess whether the algae could actively release internalized PS particles or metabolic by-products during post-exposure. Therefore, the term “PS-free medium”in this study refers strictly to the absence of suspended NPs in the bulk culture medium, rather than to the complete absence of cell-associated particles.

### Methods employed for the measurement of biochemical indicators

#### Algal cell density

The number of algal cells was quantified using microscopic counting and spectrophotometry, respectively [39]. Afterwards, the OD_680_ value and cell density were converted into a linear regression equation, as follows cell density (×10^6^ cells/mL) = 49.55 × OD_680_−10.09 (R^2^ = 0.9987). The absorbance of algal cells was determined by spectrophotometry at a wavelength of 680 nm at 48-hour intervals, and the algal density was determined using a pre-established regression equation.

#### Phycobiliprotein content

The algal cells were harvested on days 7 and 15 of exposure, and on days 4, 7, and 15 post-exposure to measure phycobiliprotein content. The concentration of phycobiliprotein was determined spectrophotometrically in accordance with previously established methodology [11], and further information was provided in the supplementary materials.

#### Oxidative stress

Microcystis cells were tested for antioxidant defense and cell damage on days 7 and 15 during exposure, as well as on days 4, 7, and 15 of post-exposure. Initially, 10 mL of the algal samples were subjected to centrifugation and, the cells were resuspended in PBS (0.05 M, pH = 7.4). The suspension was frozen three times in liquid nitrogen, then centrifuged at 8000 rpm for 10 min at 4 °C to collect the supernatant. The supernatant was analyzed for superoxide dismutase (SOD), catalase (CAT), glutathione (GSH), and malondialdehyde (MDA) levels using assay kits from Nanjing Jiancheng Bioengineering Institute, following the manufacturer’s instructions.

The overall oxidative stress status was assessed using the IBR method [30], with SOD, CAT, GSH, and MDA as biomarkers.

The following is an outline of how the IBR is determined:

1. Calculate the general mean (X) and standard deviation (S) for each biomarker.
2. Standardized the each mean X_i_: Y_i_= (X_i_- X)/ S.
3. Obtained the minimum value (Min) for all stations for each biomarker and compute the score S_i_ = Y_i_ +|Min|.
  The star plot was used to display the treatment results and Si represents the radiation length of each biomarker in the star plot.
4. Calculated the triangle areas: A_i_=0.5* S_i_*S_i _+_1_  and IBR = $\:{\sum\:}_{i=0}^{n}\mathrm{A}\mathrm{i}$.

#### Transcriptome sequencing and analysis

Analysis of physiological parameters indicated that 5 mg/L PS did not affect *M. aeruginosa* significantly. Thus, transcriptomic analysis focused solely on the 50 mg/L treatment group. Transcriptomic analysis of *M. aeruginosa* was conducted to identify differentially expressed genes (DEGs) under the defined criteria (|log₂(fold change)| ≥ 1.0; adjusted *p* ≤ 0.05). The functional implications of the DEGs were then investigated through enrichment analysis against the Gene Ontology (GO) and Kyoto Encyclopedia of Genes and Genomes (KEGG) databases. Comprehensive details on RNA extraction, library construction, sequencing, and bioinformatics are described in the supplementary materials.

#### Statistical analysis

Statistical analyses were performed using SPSS (Version 24.0, IBM), with one-way analysis of variance (ANOVA) applied for group comparisons. Data are presented as mean ± standard deviation (SD), and a *p* ≤ 0.05 was considered statistically significant. All figures were generated using Origin (Version 9.0).

## Results

### Effects of PS on the growth of *M. aeruginosa*

**Fig. 1:**
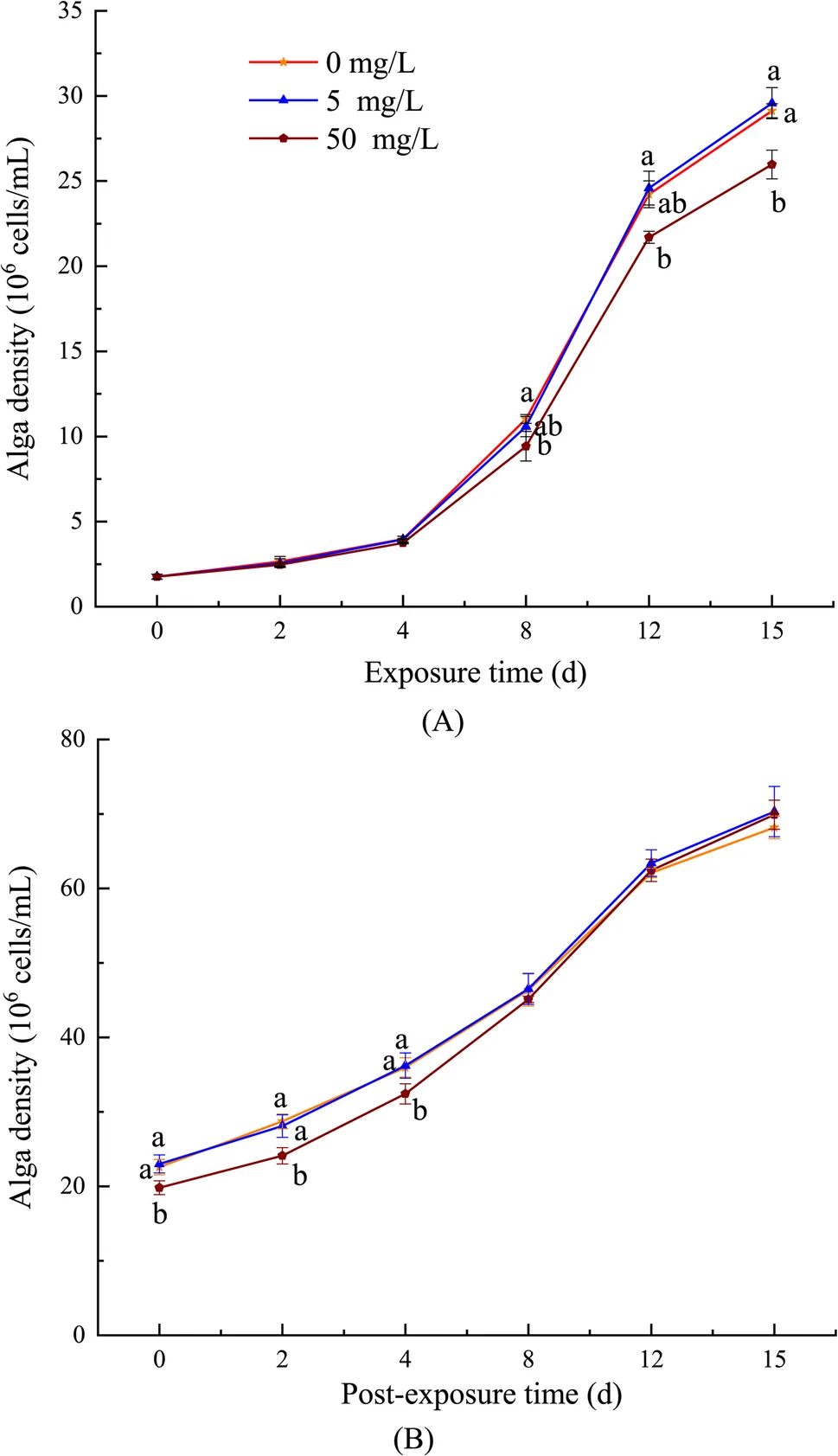
Growth dynamic of *M. aeruginosa* during PS exposure (**A**) and subsequent post-exposure periods (**B**). Vertical bars represent mean ± SD, *n* = 3. Different letters indicate significant differences among groups (*p* < 0.05)

The growth dynamics of *M. aeruginosa* over the 30-day experimental period are shown in Fig. 1. Exposure to 5 mg/L PS did not significantly affect cell density throughout the exposure phase. On day 15, cell density in this treatment (2.96 × 10⁷ cells/mL) was comparable to that of the control (2.91 × 10⁷ cells/mL). In contrast, 50 mg/L PS exhibited no notable inhibition during the initial exposure period (days 0–4), but significantly suppressed growth from day 8 to day 15, with a maximum inhibition rate of 14.53% on day 8. The inhibitory effect subsequently declined, stabilizing at approximately 10% between days 12 and 15.

During the post-exposure phase (Fig. 1B), the 5 mg/L pre-treated group showed a slight but non-significant increase in cell density relative to the control, reaching 7.03 × 10⁷ cells/mL by day 15. In contrast, the 50 mg/L pre-treated group showed significantly lower cell density on days 2 and 4, with reductions of 16.10% and 9.87%, respectively. However, by days 12 and 15, cell density reached 6.27 × 10⁷ and 6.99 × 10⁷ cells/mL, respectively, exceeding the control levels and indicating that *M. aeruginosa* exhibited a growth recovery effect during post-exposure in PS-free medium.

### Effects of PS on photosynthetic pigment content of *M.aeruginosa*

**Fig. 2:**
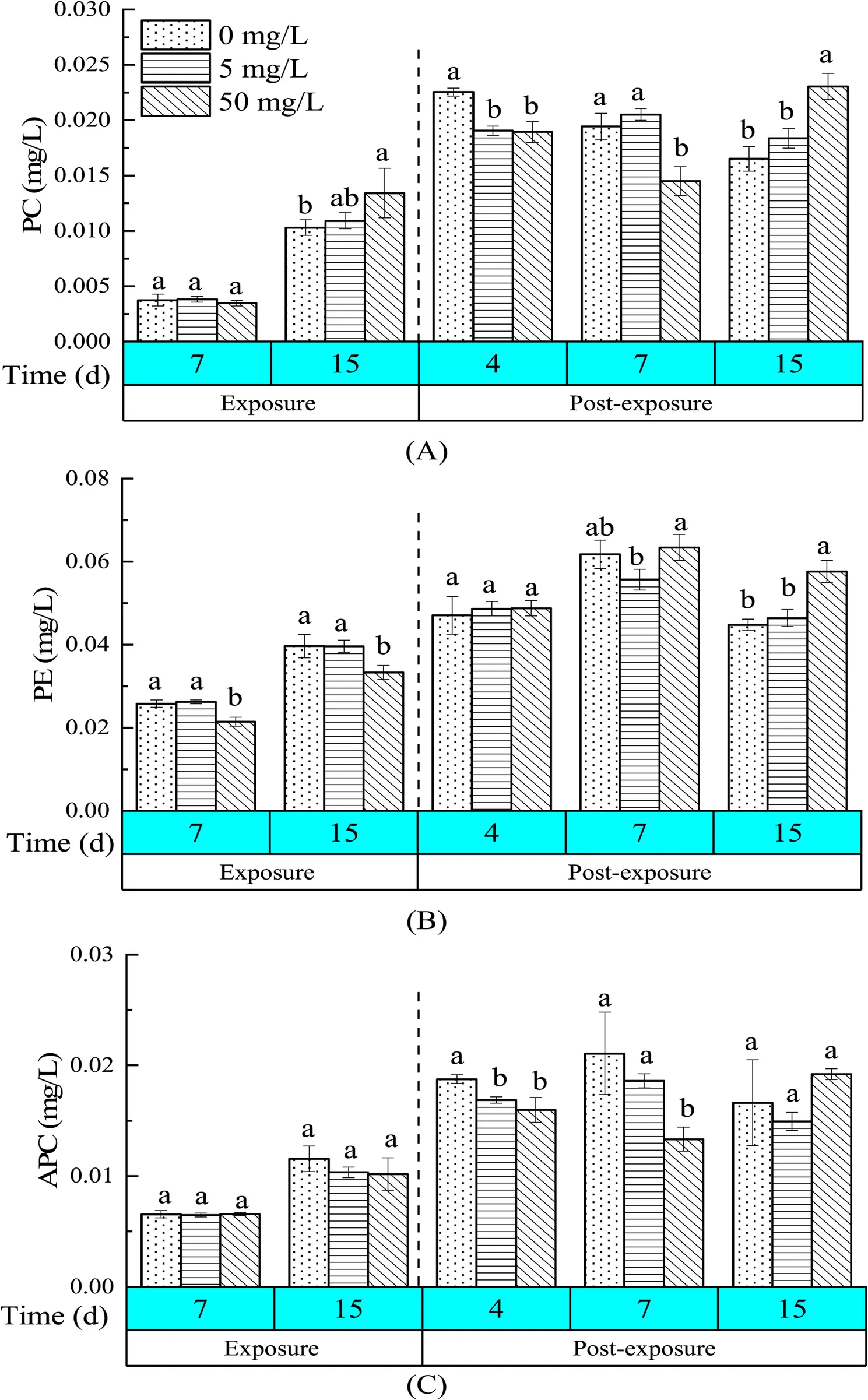
The content change of phycocyanin (**A**), phycoerythrin (**B**), allophycocyanin (**C**) in *M. aeruginosa* during PS exposure and post-exposure. Vertical bars represent mean ± SD, *n* = 3. Different letters indicate significant differences among groups (*p* < 0.05)

During exposure, no significant differences in phycocyanin (PC), phycoerythrin (PE), or allophycocyanin (APC) content were observed between the 5 mg/L PS treatment and the control. (Fig. 2A-C). In the 50 mg/L PS treatment, PE synthesis was consistently inhibited, with inhibition rates of 16.87% on day 7 and 15.98% on day 15. PC content was unaffected on day 7 but was significantly higher than the control on day 15.

In the post-exposure phase, PC and APC contents were significantly reduced in both pre-treated groups on day 4, with reductions of 15.49% and 10.13% at 5 mg/L, and 15.97% and 14.87% at 50 mg/L, respectively. By day 15, PC and APC levels in the 5 mg/L pre-treatment group had recovered to control levels, while in the 50 mg/L pre-treatment group, PC and APC were significantly higher than the control.

### Effects of PS on antioxidant stress of *M.aeruginosa*

**Fig. 3:**
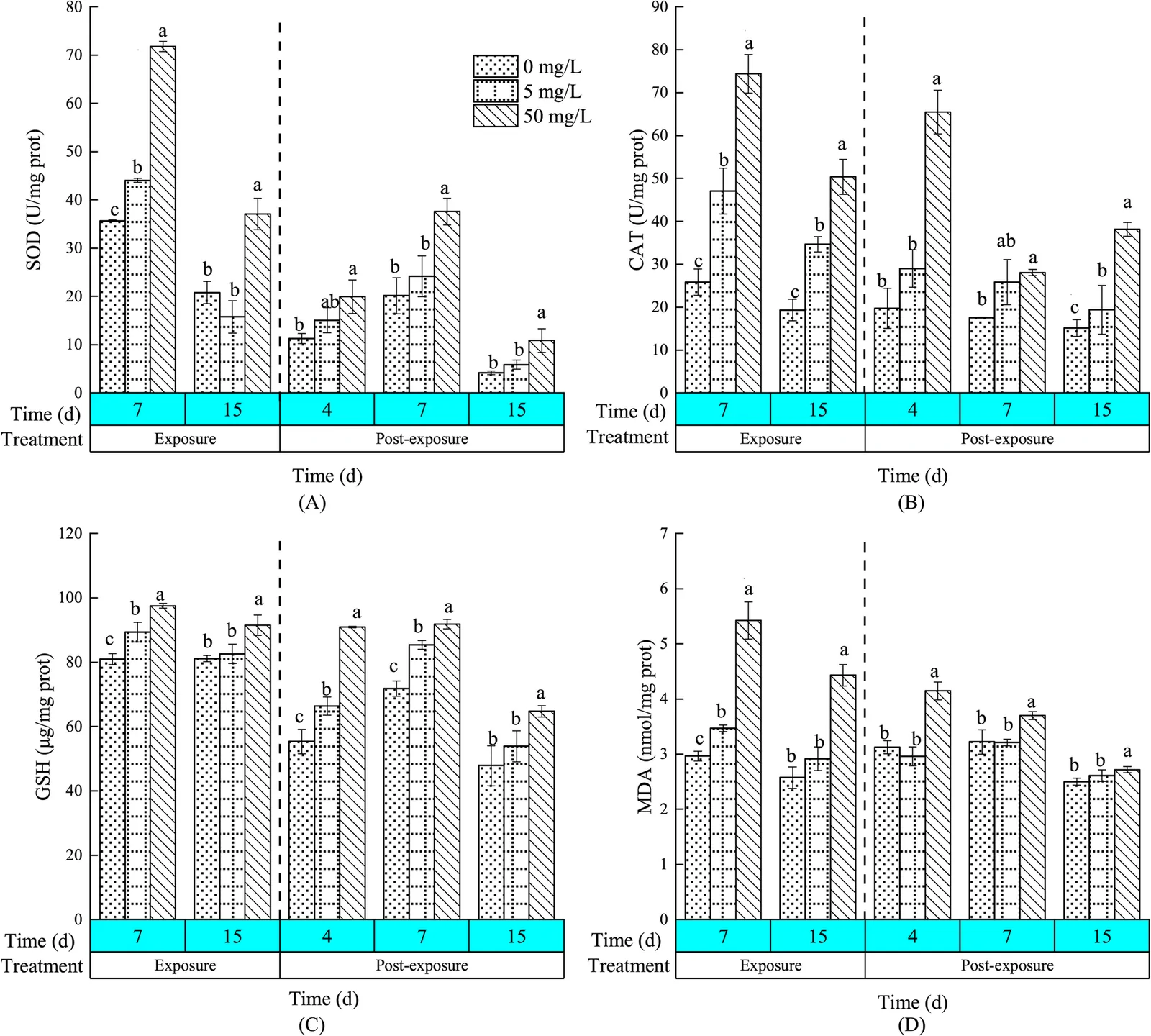
Response of *M. aeruginosa* enzymatic activities when exposed to PS and transfed to BG11 medium. The assessed indices for these tests included: (**A**) SOD, (**B**) CAT, (**C**) GSH, (**D**) MDA. Vertical bars represent mean ± SD, *n* = 3. Different letters indicate significant differences among groups (*p* < 0.05)

After 7 days of exposure, antioxidant enzyme activities were significantly elevated in a dose-dependent manner (Fig. 3A-C). As PS concentration increased from 5 to 50 mg/L, SOD and CAT activities increased from 44.02 to 71.77 U/mg prot and from 47.06 to 74.43 U/mg prot, respectively, while GSH concentration increased from 89.36 to 97.53 µg/mg prot. No significant differences in SOD activity or GSH concentration were observed between the 5 mg/L PS treatment and the control group; however, CAT activity was significantly elevated throughout the 15-day exposure period. In contrast, exposure to 50 mg/L PS resulted in sustained increases in SOD, CAT, and GSH levels, with fold changes of 1.78, 2.61, and 1.13, respectively, relative to the control on day 15.

During the post-exposure period, SOD and CAT activities in the 5 mg/L pre-treated group returned to control levels, while GSH concentration remained significantly elevated on days 4 and 7. In the 50 mg/L pre-treated group, SOD, CAT, and GSH levels were significantly induced throughout the entire post-exposure period (*p* < 0.05), with fold changes of 2.58, 2.52, and 1.35, respectively, on day 15.

MDA content was measured as an indicator of membrane damage (Fig. 3D). During exposure, MDA levels decreased over time but showed a positive correlation with PS concentration, indicating dose-dependent membrane damage. During the post-exposure period, MDA content in the 5 mg/L pre-treated group was comparable to the control, whereas the 50 mg/L pre-treated group maintained significantly higher levels throughout the post-exposure period.

### IBR analysis integrated oxidative stress response of PS on *M. aeruginosa*

The biomarkers of oxidative stress in *M. aeruginosa* exhibited disparate responses at the biochemical level following exposure to PS. It is of paramount importance to ascertain the overall oxidative stress impact of MPs on *M. aeruginosa*. The IBR is a quantitative tool for integrating a range of biomarkers to provide extensive reflection on the toxicity of MPs [30]. The standardized of MDA, SOD, CAT and GSH was conducted in this research, and the resulting data were displayed as star diagrams (Fig. S2), and Fig. S2 F displays the matching IBR values. All treatment groups and control groups showed a gradual decrease in IBR values throughout the experiment, except on day 7 of the post-exposure period. On day 15 of post-exposure, the IBR values of recorded were 0, 0.09 and 1.11 for the control, 5 and 50 mg/L PS group, respectively, while on day 15 of exposure period, the values recorded were 1.29, 2.37, 9.85. The findings indicated that PS induced oxidative stress-mediated in the exposure period can be recovered during post-exposure. Interestingly, the oxidative stress damage caused by 5 mg/L PS can be completely recovered, whereas high concentrations do not fully restore the damage.

### Transcriptome analysis

Assembly analysis generated over 6 G of raw sequence data, as detailed in Table S1 . The Q30 is a key indicator of data quality and reliability, with over 99.9% accuracy rate for the proportion of bases. In this study, Q30 of all samples was more than 92% and the proportion of clean reads that were accurately aligned with the *M. aeruginosa* reference genome was greater than 91% (Table S1), suggesting that the data quality was sufficient for further downstream analyses. Differential expression analysis identified 812 genes (341 up- and 471 down-regulated) responsive to 50 mg/L PS exposure (Fig. S3 A), while the post-exposure stage exhibited 445 DEGs, characterized by a greater number of up-regulated (266) than down-regulated (179) genes (Fig. S3 B). A total of 377 genes were identified as co-differentially expressed during the exposure and post-exposure stages (Fig. S3 C).

**Fig. 4:**
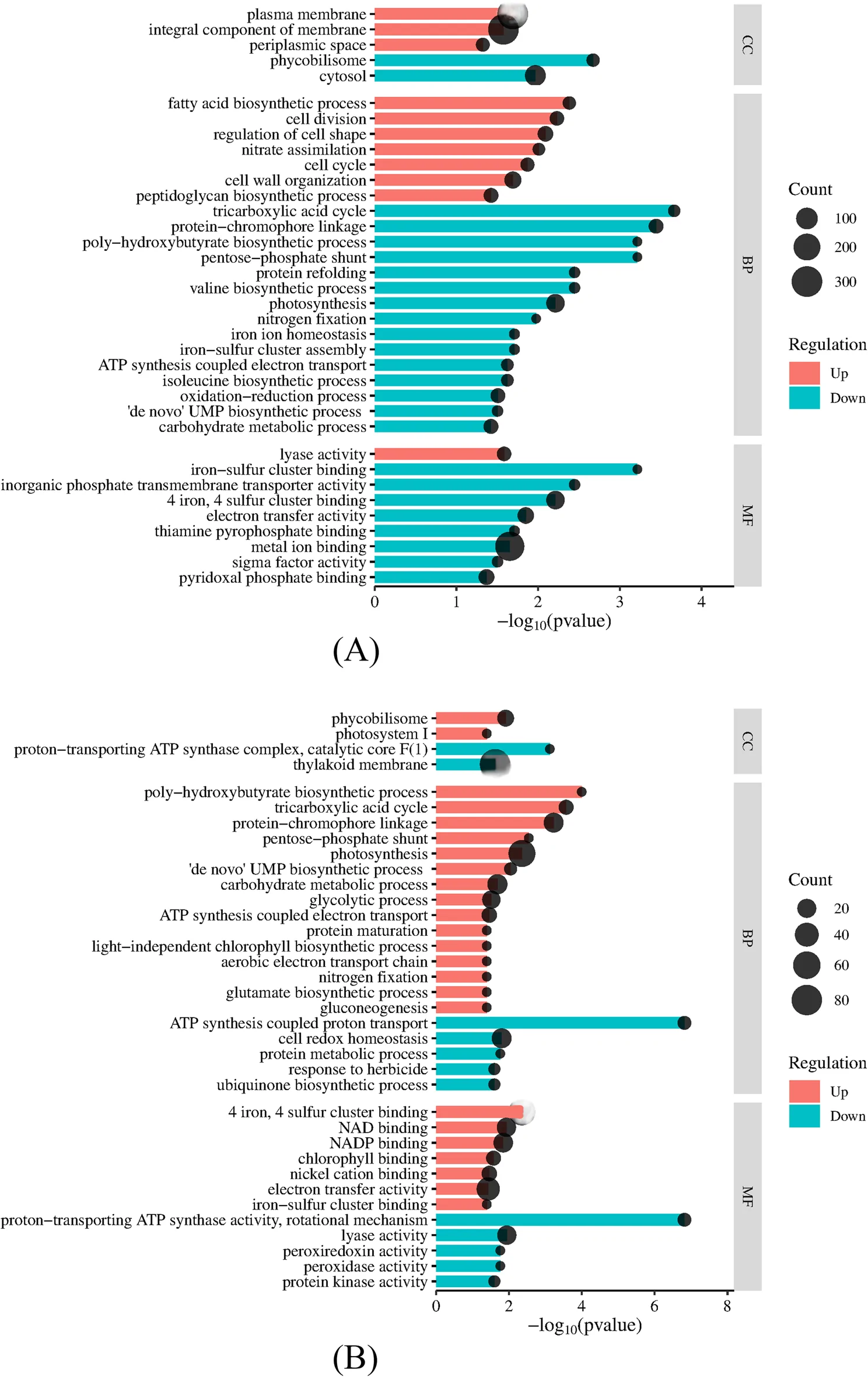
Cluster analysis of significantly enriched GO terms (*p* < 0.05) in *M. aeruginosa* following 50 mg/L PS exposure (**A**) and post-exposure. (**B**). The dotsize indicates the number of DEGs in the corresponding pathway

GO enrichment analysis revealed distinct transcriptional rebound during PS exposure and the subsequent post-exposure phase. During exposure to 50 mg/L PS, down-regulated genes were predominantly enriched in core photosynthetic and central carbon metabolic pathways, including photosynthesis, protein-chromophore linkage, and the tricarboxylic acid (TCA) cycle (Fig. 4A). Concurrently, functions associated with phycobilisome assembly, 4 iron-4 sulfur cluster binding, and electron transfer activity were also suppressed (Fig. 4A). In contrast, the up-regulated terms were primarily linked to cell structure maintenance and reproductive preparedness, with significant enrichment in plasma membrane components, cell division, cell cycle, and cell wall organization (Fig. 4A).

Following PS removal, a marked metabolic reversal was observed, with previously suppressed pathways becoming significantly up-regulated (Fig. 4B). This included post-exposure of photosynthesis, protein-chromophore linkage, phycobilisome, and electron transfer activity (Fig. 4B). The TCA cycle, along with associated pathways such as glycolytic process and pentose-phosphate shunt, were also up-regulated (Fig. 4B). Notably, the polyhydroxybutyrate (PHB) biosynthetic process was highly induced. Enhanced glutamate biosynthesis and nitrogen fixation were also detected, along with up-regulation of de novo UMP biosynthesis (Fig. 4B).

**Fig. 5:**
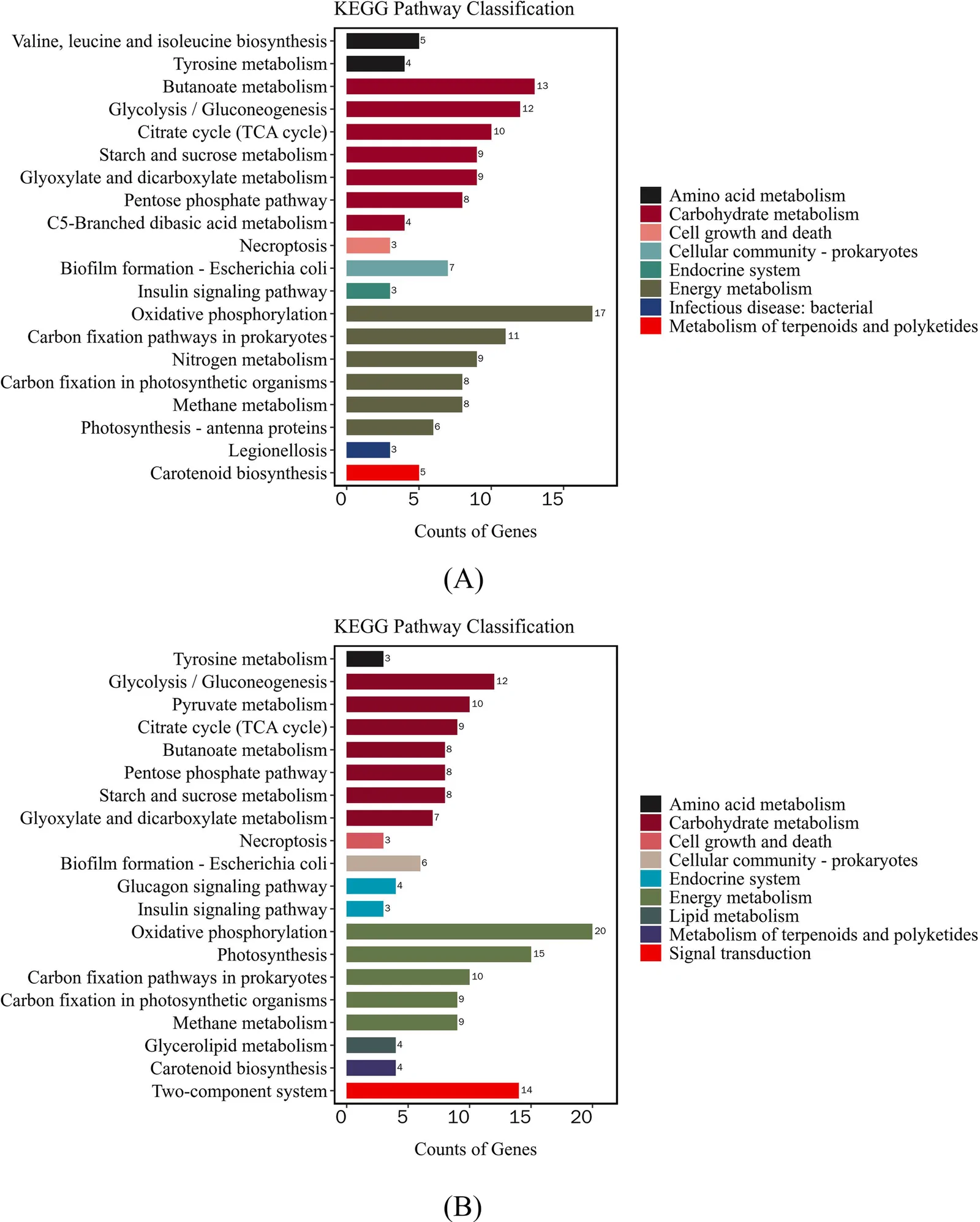
The top 20 Kyoto Encyclopedia of Genes and Genomes (KEGG) pathways during 50 mg/L PS exposure (**A**) and post-exposure (**B**)

KEGG pathway enrichment analysis was conducted to investigate the molecular mechanisms underlying PS exposure and post-exposure. The top 20 enriched pathways were predominantly associated with carbohydrate and energy metabolism in both exposure and post-exposure stages (Fig. 5).During exposure to 50 mg/L PS, genes involved in glycolysis were markedly downregulated, including 6-phosphofructokinase (PFK, log₂FC = -2.97), glyceraldehyde-3-phosphate dehydrogenase (GAPDH, log₂FC = -4.11), and pyruvate kinase (PK, log₂FC = -2.97). Concurrently, TCA cycle genes were suppressed, as evidenced by the downregulation of malate dehydrogenase (MDH, log₂FC = -2.07), succinate dehydrogenase (SDHB, log₂FC = -2.86), and citrate synthase (CS, log₂FC = -1.74) (Table 1). In the post-exposure period, this expression pattern was reversed, with most previously suppressed genes showing significant upregulation. GAPDH and PK exhibited log₂FC values of 3.33 and 1.73, respectively, while TCA cycle genes such as SDHB (log₂FC = 2.05) and NADP-dependent isocitrate dehydrogenase (Idh2, log₂FC = 1.65) were also induced (Table 1).

The pentose phosphate pathway (PPP) displayed a pronounced temporal reconfiguration (Fig. 5; Table 1). During PS exposure period, the oxidative phase was initially suppressed, as evidenced by downregulation of rate-limiting enzyme glucose-6-phosphate dehydrogenase (G6PD, log₂FC = -2.56) and 6-phosphogluconate dehydrogenase (PGD, log₂FC = -4.39). In contrast, these genes were upregulated during post-exposure, with log₂FC 3.00 and 4.25, respectively (Table 1). Additionally, xylulose-5-phosphate/fructose-6-phosphate phosphoketolase (Xpk, LRR78_RS13295) was strongly upregulated during post-exposure (log₂FC = 3.00).

**Table 1:** Differently expressed genes related to glycolysis/gluconeogenesis, TCA cycle and pentose phosphate pathway

Gene_ID	Annotation	log₂ ^(fold change)^	
		PS50-15 VS CK15	PS50-30 VS CK30
**Glycolysis/gluconeogenesis**			
**LRR78_RS15705**	Glucokinase	-1.33	1.20
LRR78_RS19325	Fructose-1,6-bisphosphatase I	-1.95	1.23
LRR78_RS11315	6-phosphofructokinase	-2.97	2.42
LRR78_RS14220	Triosephosphate isomerase	1.23	-1.19
LRR78_RS13300	Glyceraldehyde 3-phosphate dehydrogenase	-4.11	3.33
LRR78_RS14375	Glyceraldehyde-3-phosphate dehydrogenase(NAD(P)+)	1.10	-1.11
**LRR78_RS02850**	2,3-bisphosphoglycerate-independent phosphoglycerate mutase	1.04	-
LRR78_RS02965	Enolase	-2.15	1.90
LRR78_RS22045	Pyruvate kinase	-2.97	1.73
LRR78_RS05450	Fructose-bisphosphate aldolase, class I	-	-1.14
**TCA cycle**			
LRR78_RS03465	Isocitrate/isopropylmalate dehydrogenase	-1.41	1.72
LRR78_RS05000	Malate dehydrogenase	-2.07	1.07
LRR78_RS09000	Succinate dehydrogenase	-1.69	-
LRR78_RS10030	Succinate dehydrogenase	-2.86	2.05
LRR78_RS14040	Citrate synthase	-1.74	1.34
LRR78_RS16625	Nadp-dependent isocitrate dehydrogenase	-1.72	1.65
LRR78_RS22825	Heterodisulfide reductase	-2.93	2.28
LRR78_RS08700	Aconitate hydratase	-1.07	-
LRR78_RS13750	Fumarate hydratase	-	1.16
**Pentose phosphate pathway**			
LRR78_RS23185	Glucose-6-phosphate 1-dehydrogenase	-2.56	3.00
LRR78_RS14240	6- phosphogluconolactonase	-1.57	-
LRR78_RS01905	6-phosphogluconate dehydrogenase	-4.39	4.25
LRR78_RS07115	Ribulose-phosphate 3-epimerase	2.18	-1.94
LRR78_RS12565	Transaldolase	-1.48	1.73
LRR78_RS13295	Xylulose-5-phosphate/fructose-6-phosphate phosphoketolase	-2.95	3.00

Oxidative phosphorylation was the most significantly enriched pathways in energy metabolism (Fig. 5). Key genes involved in this pathway were markedly downregulated during PS exposure, but exhibited significant upregulation during the post-exposure phase (Fig S4). These genes fall into three major categories: NADH dehydrogenase (NdhE, HoxE, HoxF, HoxU), succinate dehydrogenase (SdhC, SdhA, SdhB), and cytochrome c oxidase (CyoE, CoxA, CoxB, CoxC, CydA, CydB, COX10, COX15).

## Discussion

### Integrated growth inhibition, photosynthetic impairment, and oxidative stress in *M. aeruginosa* under PS exposure and post-exposure

The impact of PS on *M. aeruginosa* exhibited clear temporal and concentration-related dynamics in this study. This concentration-dependent toxicity is commonly observed in MPs-algae interactions. For instance, 1–5 mg/L PS did not significantly affect the growth of *Chlorella vulgaris*, whereas 50–1000 mg/L markedly suppressed it [35]. Similarly, polyethylene (PE) also inhibited the growth and chlorophyll-a synthesis in *Chlorella* in a dose-dependent manner [37]. At low concentrations, MPs may induce adaptive responses in algae, allowing maintenance of normal growth. In contrast, at elevated concentrations, they can form a physical barrier on cell surfaces, impairing nutrient uptake and gas exchange [27]. Additionally, MPs particles can adhere to algal cells, reducing light penetration and compromising photosynthetic efficiency, which subsequently leads to reduced pigment content and growth suppression [39].

The algae density showed a moderate increase in the 5 mg/L P post-exposure period, potentially attributable to hormesis—a phenomenon in which exposure to low-level stressors stimulates beneficial adaptive responses [18]. The findings underscore a pronounced resilience in *M.aeruginosa*, as manifested by the reduced inhibition at 50 mg/L PS and the complete post-exposure growth post-exposure. The observed post-exposure likely stems from metabolic adaptation and active defense mechanisms, enabling the cyanobacteria to mitigate and overcome the transient stress induced by NPs [21]. Phycobiliproteins are crucial pigment-protein complexes in cyanobacteria for harvesting light energy, playing a vital role in capturing photons and transferring energy to the photosynthetic reaction centers [28]. After 15 days of exposure, the phycobiliprotein content in the 50 mg/L PS treatment group increased significantly, followed by elevated levels of PC and APC during the post-exposure phase (Fig. 2). These findings suggest that microalgae dynamically regulate the content and composition of phycobiliproteins to optimize photosynthetic efficiency, thereby enabling cyanobacteria to adapt to PS-induced stress and resume growth [15].

The antioxidant system of *M. aeruginosa* was markedly activated by PS exposure, with the intensity and duration of response being greater at 50 mg/L (Fig. 3A-C). Numerous studies indicate that exposure to MPs triggers oxidative stress and associated toxic effects in microalgae [18]. The elevated activities of SOD, CAT, and GSH confirm the generation of reactive oxygen species (ROS). Excessive ROS accumulation adversely affects the synthesis of chlorophyll a and other photosynthetic pigments (Fig. 2), thereby constraining the ability of algal cells to capture and convert light energy [5]. Moreover, the interaction of ROS with cell membrane phospholipids leads to lipid peroxidation and increased MDA content (Fig. 3D), thereby affecting membrane’s fluidity, permeability, structural integrity, and functional capacity [40]. In the 5 mg/L PS group, oxidative stress was subsequently alleviated, likely due to the activation of adaptive mechanisms [42]. In contrast, sustained oxidative stress was observed at 50 mg/L PS (Fig. 3), possibly due to limited damage repair capacity under persistent or high-intensity pollution [4].

### Transcriptional dynamics in *M. aeruginosa* during PS exposure and the subsequent post-exposure phase

GO enrichment analysis demonstrated significant inhibition of photosynthesis, protein-chromophore linkage, and phycobilisome in 50 mg/L PS exposure (Fig. 4A). As the key antenna complex for light energy capture in cyanobacteria, the loss of phycobilisome directly severs the primary energy source of cells, exerting profound impacts on cellular energy metabolism [48]. Concurrently, KEGG pathway analysis confirmed coordinated downregulation of glycolysis (PFK, GAPDH, PK) and the TCA cycle (MDH, SDHB, CS) (Table 1; Fig. 5A). Notably, IDH3 catalyzes the oxidative decarboxylation of isocitrate to α-ketoglutarate, coupled with NADH production. The observed downregulation of IDH3 suggests that *M.aeruginosa* adapts to 50 mg/L PS exposure by attenuating non-essential energy-yielding processes. You et al., [45] have also found that the main down-regulated pathway was TCA cycle when *Synechocystis* sp. exposed to the 50 μm PS. During the post-exposure phase, following the mitigation of PS-induced stress, a marked upregulation was observed in both photosynthetic capacity and key central carbon metabolism pathways, such as the TCA cycle and glycolysis. (Fig. 5B; Table 1). Increasing these carbon metabolic pathways allows algal cells to regulate the body’s ATP and NADH, ensuring the body’s energy supply. This rebound not only compensates for the depleted energy reserves (such as ATP) during the stress period but is also likely to provide ample carbon skeletons and energy for large-scale repair and reconstruction processes [44].

Notably, cells exhibit a non-passive response to the energy crisis during 50 mg/L PS exposure. We observed a marked induction of genes involved in plasma membrane integrity, cell division, and cell wall organization (Fig. 4A). Both NPs and MPs could disrupt *M.aeruginosa* cellular integrity, via membrane adsorption, rupture, and deformation by NPs, or membrane thickening and chloroplast damage by MPs [47]. Therefore, in this study, the upregulation of plasma membrane-related genes in microalgae may contribute to enhanced plasma membrane integrity and function, thereby mitigating the adverse effects of PS. The cell wall, with its highly diverse composition and architecture, is fundamental to microalgal survival and environmental adaptation [13]. Evidence indicates that exposure to aluminum oxide nanoparticles induces cell wall damage in *Chlorella* [29]. Furthermore, a analogous thickening response has been observed in *Chlorella* exposed to PS [21], which is implicated in blocking MPs intrusion and mitigating subsequent cellular damage. In the present study, the upregulation of cell wall organization-related genes suggests an active reinforcement and structural modification of the cell wall in response to MPs stress. This structural adaptation likely entails the synthesis of nascent cell wall components and/or the reconfiguration of existing ones [10], collectively functioning to enhance resistance against NPs. Cell division is an indispensable process for the growth and dispersal of microalgae in the environment [46]. Existing studies have indicated that low concentrations of BPAF and PFOA promote cell division in *M. aeruginosa* [24]. In summary, microalgae upregulate genes associated with the plasma membrane, cell division, and cell wall organization during energy crises, reflecting an active defense and maintenance strategy under extreme environmental conditions.

The dynamic change of the PPP is particularly noteworthy in the microalgal oxidative stress response. During the exposure phase, the oxidative branch of the PPP responsible for generation NADPH was significantly suppressed. Conversely, in the post-exposure phase, the expression of key PPP enzymes—including G6PD and PGD—was markedly upregulated (Table 1), leading to a rapid and robust supply of NADPH. As an essential reductant cofactor in antioxidant systems, particularly the glutathione cycle, NADPH effectively scavenges reactive oxygen species and mitigates oxidative damage [38]. Concurrently, upregulation of the non-oxidative PPP gene, Xpk, promotes the synthesis of ribose-5-phosphate, a critical precursor for nucleotide biosynthesis [33]. The enhanced nucleotide synthesis facilitates cellular repair of damaged DNA/RNA and supplies necessary macromolecules for the proliferation and replacement of impaired cells, thereby restoring the accuracy of genetic information and cellular functions [33]. The active state of the entire PPP not only provides NADPH and nucleotide precursors but is also closely linked to the rebound of overall cellular energy metabolism. The PPP shares intermediates with the glycolytic pathway, and this cross-complementarity enables cells to flexibly allocate carbon flux according to specific demands [16]. Collectively, the coordinated regulation of the PPP’s oxidative and non-oxidative phases facilitates the restoration of genetic integrity and cellular function in microalgae under oxidative stress. Furthermore, there was an upregulation of biosynthesis processes characteristic of the post-exposure phase, such as PHB and de novo UMP synthesis in this study (Fig. 4B). As a common carbon storage compound, the accumulation of PHB may serve as a strategic reserve established to cope with potential recurring stress [25]. Meanwhile, the enhancement of nucleotide synthesis reflects the cellular preparation necessary for entering a new growth cycle [17]. Therefore, the upregulation of these biosynthetic pathways not only aids microalgae in surviving unfavorable conditions but also lays the foundation for subsequent growth and proliferation.

Among the energy metabolism pathways, oxidative phosphorylation exhibited the most profound impact (Fig.S4). Mitochondria generate the majority of cellular energy (ATP) through the oxidative phosphorylation system, while also producing ROS [14]. NADH dehydrogenase (Complex I) and cytochrome c oxidase (Complex IV) are key components of the electron transport chain in oxidative phosphorylation. During PS exposure periods, when TCA cycle activity is reduced, NADH production correspondingly decreases. Under such conditions, by downregulating the expression of critical ETC-related genes, cells can effectively reduce the quantity of ETC complex proteins, thereby minimizing electron leakage and avoiding more severe oxidative damage [36]. In contrast, the reactivation of the TCA cycle during post-exposure provides abundant NADH. Cells respond by synergistically upregulating core ETC enzymes such as NADH dehydrogenase and cytochrome c oxidase, thereby restoring efficient energy transduction. This metabolic adaptation delivers the necessary energy to power critical repair mechanisms—e.g., degradation and synthesis of damaged proteins, membrane repair, and cell proliferation—while supporting biosynthetic pathways for macromolecule production. However, antioxidant enzyme activities (SOD, CAT, GSH) and MDA levels remained elevated throughout this period (Fig. 3). This apparent contradiction likely reflects incomplete resolution of oxidative stress. Enhanced electron transport chain activity, while increasing ATP synthesis, may also promote electron leakage and continued ROS generation [36]. Persistently elevated antioxidant enzymes appear to compensate for this ongoing pro-oxidant state, while sustained MDA levels indicate unresolved membrane damage. Therefore, the post-exposure phase does not represent a complete return to pre-exposure conditions, but rather a new redox homeostasis with elevated energy metabolism and persistent oxidative stress.

The observed effects in this study likely stem from the combined action of the physical characteristics (e.g., small size) and chemical composition of the NPs. It should be noted that without a MPs control of identical chemical composition, it is not possible to precisely quantify the contribution of size-specific effects alone. Nevertheless, existing literature suggests that NPs typically exhibit greater biological effects than their larger counterparts [39]. To further elucidate the relative contributions of pure size effects versus chemical composition to the toxicological impact of NPs on *M. aeruginosa*, future research could employ particles of varying sizes but identical chemical composition, or particles of similar size with differing chemical compositions. Such controlled comparative studies would enable the quantification of each factor’s independent contribution.

It should be noted that the BG11 culture medium employed in this study possesses a relatively high ionic strength, which may facilitate the aggregation of PS over the 15-day exposure period [6]. Such aggregation increases the effective particle size of the PS, thereby accelerating their sedimentation rate. Moreover, the accompanying change in particle size can alter the mode of interaction between NPs and organisms, as well as the underlying toxicity mechanisms [22]. In the present study, particle size and stability were characterized only at the beginning of the experiment (Fig. S1), and we did not monitor the aggregation state or sedimentation process throughout the exposure and post-exposure phases. Therefore, the observed toxic effects and subsequent post-exposure likely result not solely from dispersed NPs but from the combined action of dispersed nanoparticles, larger aggregates, and the settled phase. Future studies should employ real-time particle characterization techniques or more environmentally relevant exposure systems (e.g., natural waters with lower ionic strength) to better elucidate the contribution of particle aggregation to NPs toxicity.

## Conclusions

This study integrated physiological, biochemical, and transcriptomic study demonstrated that PS exert concentration-related effects on *M. aeruginosa*, with the cyanobacterium exhibiting a remarkable recovery capacity during post-exposure. The key findings are:

1. Low-concentration impact: Exposure to 5 mg/L PS induced only mild oxidative stress but did not significantly inhibit algal growth, photosynthetic pigment synthesis, or central metabolic pathways. Physiological post-exposure was rapid and complete upon the removal of the stressor.
2. High-concentration toxicity: Exposure to 50 mg/L PS significantly suppressed algal growth by broadly disrupting core energy metabolism, including photosynthesis, glycolysis, the TCA cycle, and oxidative phosphorylation, thereby inducing substantial oxidative stress.
3. Metabolic post-exposure: The removal of PS triggered a metabolic rebound. The robust upregulation of previously suppressed pathways, particularly the NADPH-generating pentose phosphate pathway and oxidative phosphorylation, was identified as the key mechanism driving the substantial restoration of physiological functions. However, persistent oxidative stress and membrane damage indicated that this post-exposure recovery remained incomplete by the end of the experimental period.

## Supplementary Information

Supplementary Material 1

## Data Availability

The raw sequence data reported in this paper have been deposited in the Genome Sequence Archive (GSA) at the National Genomics Data Center, China National Center for Bioinformation / Beijing Institute of Genomics, Chinese Academy of Sciences, under accession number CRA041727, and are publicly accessible at https://ngdc.cncb.ac.cn/gsa. All other data generated or analyzed during this study are included in this published article and its supplementary information files.

## Abbreviations

APC: Allophycocyanin
CAT: Catalase
CS: Citrate synthase
DEGs: Differentially expressed genes
G6PD: Glucose-6-phosphate dehydrogenase
GAPDH: Glyceraldehyde-3-phosphate dehydrogenase
GO: Gene Ontology
GSH: Glutathione
Idh2: NADP-dependent isocitrate dehydrogenase
KEGG: Kyoto Encyclopedia of Genes and Genomes
*M. aeruginosa*: *Microcystis aeruginosa*
MDA: Malondialdehyde
MDH: Malate dehydrogenase
MPs: Microplastics
NPs: Nanoplastics
PC: Phycocyanin
PE: Phycoerythrin
PFK: Phosphofructokinase
PGD: 6-phosphogluconate dehydrogenase
PHB: Polyhydroxybutyrate
PK: Pyruvate kinase
PPP: Pentose phosphate pathway
PS: Polystyrene
ROS: Reactive oxygen species
SDHB: Dehydrogenase
SOD: Superoxide dismutase
TCA: Tricarboxylic acid
Xpk: Xylulose-5-phosphate/fructose-6-phosphate phosphoketolase
