# Supplementary material for: From inhibition to recovery: metabolic rebound of Microcystis aeruginosa following pulse exposure to polystyrene nanoplastics
Source: BMC Microbiol. 2026 Jun 8;26:747. doi: 10.1186/s12866-026-05260-9 (PMC13491635; doi:10.1186/s12866-026-05260-9)
Supplement: Supplementary file 1 [file 12866_2026_5260_MOESM1_ESM.docx]

**Phycobiliprotein Content**

In order to determine the concentration of phycobiliprotein of *M. aeruginosa*, 5 mL of algal samples were separated by centrifugation at 8000×g at 4°C for 15 minutes. Then the algal cells were resuspended in phosphate-buffered saline (PBS) and repeatedly frozen in liquid nitrogen three times. Subsequently, the supernatant was isolated by centrifugation at 10,000×g for 10 min and measured at 565 nm, 620 nm, and 650 nm, respectively. Finally, the contents of phycocyanin, phycoerythrin, allophycocyanin and total phycobiliproteins were calculated as follows:

C_PC_ = (A_620nm_ - 0.7 × A_650nm_) / 7.38

C_APC_ = (A_650nm_ - 0.19 × A_620nm_) / 5.65

C_PE_ = (A_565nm_ - 2.8 × C_PC_ - 1.34 × C_APC_) / 1.27

C_T_ = C_PC_ + C_APC_ + C_PE_

where C_PC_, C_APC_, C_PE_ and C _Phycobiliproteins_ were the concentrations of phycocyanin, phycoerythrin, allophycocyanin and total phycobiliproteins, respectively.

**Transcriptome Sequencing and Analysis**

Total RNA was extracted from *M. aeruginosa* at the end of exposure and post-exposure periods, using the mirVana™ miRNA Isolation Kit (Invitrogen). RNA integrity was verified by agarose gel electrophoresis and an Agilent 2100 Bioanalyzer (Agilent Technologies, Santa Clara, CA, USA). RNA-Seq libraries were constructed with the VAHTS Universal V6 RNA-seq Library Prep Kit and sequenced on an Illumina Novaseq 6000 platform (OE Biotech, Shanghai, China). Gene expression levels were quantified via the RPKM method, and differentially expressed genes (DEGs) were identified using DESeq2 under the criteria of |log₂ (fold change)| ≥ 1.0 and an adjusted P-value ≤ 0.05. Functional annotation and enrichment analysis of the DEGs were subsequently conducted using the Gene Ontology (GO) and Kyoto Encyclopedia of Genes and Genomes (KEGG) databases

Table S1 Transcriptome profiles of the *M. aeruginosa* exposed to 50 mg/L PS-NPs.

| Sample | Raw Bases  (G) | Clean Bases  (G) | Valid Bases (%) | Q30  (%) | Total mapped  (%) |
| --- | --- | --- | --- | --- | --- |
| CK1-15 | 6.95 | 6.87 | 98.81 | 92.67 | 96.43 |
| CK2-15 | 6.76 | 6.68 | 98.82 | 93.19 | 93.70 |
| CK3-15 | 6.83 | 6.62 | 96.93 | 92.89 | 95.20 |
| 50-1-15 | 6.75 | 6.66 | 98.71 | 93.42 | 92.70 |
| 50-2-15 | 7.12 | 6.31 | 88.53 | 93.07 | 91.55 |
| 50-3-15 | 6.96 | 6.20 | 89.09 | 92.82 | 93.26 |
| CK1-30 | 6.53 | 6.36 | 97.45 | 93.66 | 95.05 |
| CK2-30 | 7.72 | 7.2 | 93.25 | 93.51 | 93.50 |
| CK3-30 | 6.23 | 6.15 | 98.76 | 92.11 | 93.76 |
| 50-1-30 | 7.85 | 7.35 | 93.61 | 92.16 | 96.60 |
| 50-2-30 | 7.25 | 7.15 | 98.7 | 93.17 | 95.39 |
| 50-3-30 | 7.34 | 7.25 | 98.8 | 93.51 | 94.97 |

Fig.S1 Size distribution analysis of PS microspheres

Fig. S2 Star plot for the IBR of biochemical levels of M. aeruginosa when exposed to PS-NPs on the 7th (A), 15th (B), and transfed to BG11 medium on the 4th (C), 7th (D)and 15th day (E). IBR value response model of M. aeruginosa under different PS-NPs doses and exposure times (F).

(A)
 (B)

(C)

Fig.S3.Volcano plot of differentially expressed gene (DEGs) during PS-NPs exposure (A) and post-exposure (B); (C) Venn diagram showing the number of the same DEGs and unique DEGs between the exposure and post-exposure periods

(A)

(B)

Fig.S4 Heat map of key genes participated in oxidative phosphorylation pathway under exposure (A) and post-exposure (B).. Red:upregulation; Green: downregulation.. Oring:upregulation; Blue: downregulation.
